# Intracranial Tuberculoma Mimicking Neurosarcoidosis: A Clinical Challenge

**DOI:** 10.3390/idr13010020

**Published:** 2021-03-01

**Authors:** Fatemah Abbasi, Muhammet Ozer, Kirti Juneja, Suleyman Yasin Goksu, Babak Jamasian Mobarekah, Marc S. Whitman

**Affiliations:** 1 Department of Internal Medicine, Capital Health Regional Medical Center, Trenton, NJ 08638, USA; fabbasi@capitalhealth.org (F.A.); Bjamasian@capitalhealth.org (B.J.M.); 2 Bharati Vidyapeeth Deemed University Medical College, Pune, Maharashtra 411043, India; kirtijuneja89@gmail.com; 3 Department of Internal Medicine, University of Texas Southwestern Medical School, Dallas, TX 75390, USA; syasin.goksu@gmail.com; 4 Division of Infectious Diseases, Capital Health Regional Medical Center, Trenton, NJ 08638, USA; mwhitman@capitalhealth.org

**Keywords:** intracranial tuberculoma, neurosarcoidosis, non-caseous granuloma, AFB culture, brain biopsy

## Abstract

Central nervous system (CNS) tuberculosis is a rare manifestation of all tuberculosis presentations. The incidence of brain tuberculoma is increasing in developed countries due to HIV infection and immigration from tuberculosis-endemic countries. Symptoms and radiologic findings of CNS tuberculosis can be non-specific and lead to misdiagnosis or mistreatment. Intracranial tuberculoma can present with a seizure, intracranial hypertension, or focal neurologic symptoms. In our case, the diagnosis was challenging between neurosarcoidosis and intracranial tuberculoma due to inconclusive results of stereotactic brain biopsy and clinical presentation. The pathology result of the open brain biopsy revealed non-caseating granuloma. Finally, we were able to diagnose intracranial tuberculoma following acid-fast bacilli culture results of open brain biopsy. This report highlights the importance of including intracranial tuberculoma in the differential diagnosis of cerebral space-occupying lesions, even in patients with negative laboratory findings of tuberculosis.

## 1. Introduction

Central nervous system (CNS) disease caused by *Mycobacterium tuberculosis* is a rare yet highly devastating extra-pulmonary manifestation of tuberculosis. CNS tuberculosis constitutes approximately 1% of all tuberculosis cases and is associated with high mortality and morbidity [1]. The incidence of brain tuberculoma is increasing in developed countries due to HIV infection prevalence and immigration from tuberculous endemic countries. CNS tuberculosis is five times more frequent in patients with HIV infection [2]. The usual manifestations of CNS tuberculosis include tuberculous meningitis, which is the most common, followed by intracranial tuberculoma and tuberculous abscess. Salaskar et al. reported that approximately 4% of the central nervous system lesions are seen in developed countries and are 15–30% seen in developing countries, caused by tuberculosis [3].

Clinical presentation and radiologic findings of intracranial tuberculomas are mainly non-specific, which makes diagnosis challenging. Headache, seizures, altered mental status, and focal neurological deficits are common presentations of intracranial tuberculomas that can be associated with many other cerebral expansive lesions. Furthermore, findings from imaging techniques such as computerized tomography (CT)of the head and magnetic resonance imaging (MRI) of the brain are neither confirmatory nor specific for intracranial tuberculomas [4]. Intracranial tuberculomas can be located at any site within the brain parenchyma, and they may be solitary or multiple. Tuberculoma has varied MRI findings, depending on the maturation stage, i.e., it can appear as non-caseating, caseating with a solid center, or caseating with a liquid center [5].

In the current literature, up to 14% of intracranial tuberculomas can present with non-caseating granulomas [6]. Sarcoidosis is a multi-systemic disorder characterized by the presence of non-caseating granulomas. Sarcoidosis can affect any portion of the central or peripheral nervous system. Patients with brain and spinal cord involvement can present with cognitive or behavioral problems and/or focal neurologic deficits referable to the anatomic area involved. Thus, tuberculosis and sarcoidosis can present a diagnostic dilemma as they have overlapping clinical and radiological features [7]. Here, we describe a case of intracranial tuberculoma in a patient who was thought to have a neuro-sarcoidosis. The non-specific nature of the symptoms and radiological findings may lead to misdiagnosis and mistreatment of intracranial tuberculomas. Therefore, it is essential to consider intracranial tuberculomas as a differential diagnosis.

## 2. Case Presentation

**Figure 1:**
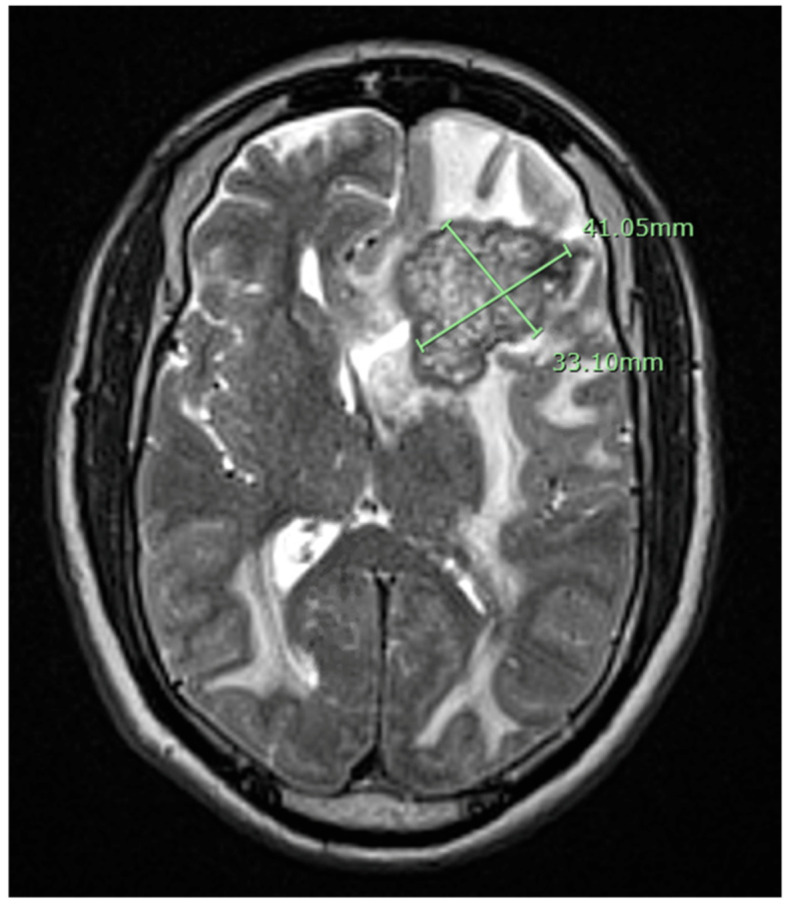
The magnetic resonance imaging (MRI) of the brain with gadolinium contrast showed a 41 mm × 33 mm heterogeneous peripherally enhancing lesion in the left frontal lobe with vasogenic edema. It shows mass effect including 13 mm of left to right midline shift and compression of the left frontal horn.

**Figure 2:**
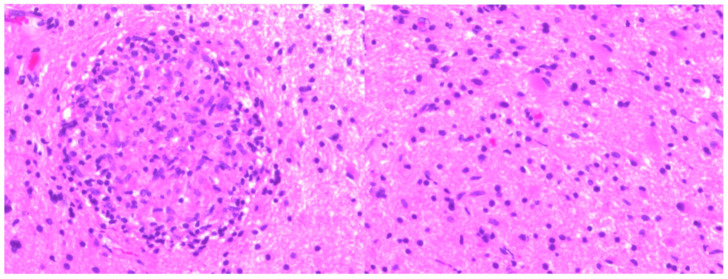
Microscopic sections demonstrate abundant epithelioid non-caseous granulomas (CD68 immunostain) associated with focal mixed chronic inflammation (CD3, CD43, and CD20) involving brain parenchyma.

**Figure 3:**
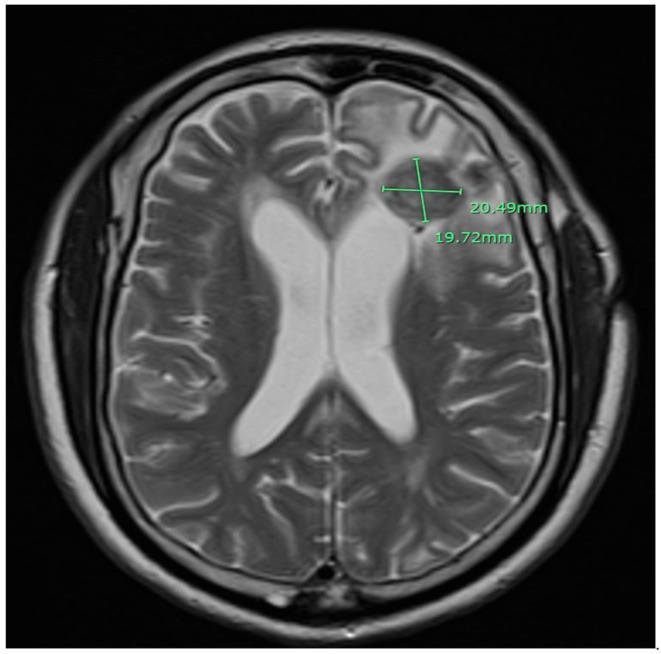
Control magnetic resonance imaging (MRI) of the brain with gadolinium showed 20 mm × 19 mm left frontal lobe mass is significantly decreased in size relative to the previous examination and does not demonstrate the significant mass effect.

A 79-year-old African American male patient with a past medical history of prostate cancer presented with progressive weakness, headache, and confusion. Brain MRI with gadolinium contrast showed a 41 × 33 × 41 mm left frontal lesion at the surface of the left lateral ventricle with vasogenic edema and midline shift (Figure 1). A stereotactic biopsy of the lesion presented the possibility of a non-caseating granuloma. Metastatic prostate cancer was ruled out. The free and total prostate specific antigen (PSA) level was within the normal limit. The patient had immigrated from Liberia but denied any constitutional symptoms such as fever, cough, night sweats, or weight loss. Quantiferon gold test was positive but active pulmonary tuberculosis was ruled out with chest imaging. The basic metabolic panel was unremarkable; complete blood count showed lymphocytosis only. HIV antibodies were negative. Subsequently, we proceeded with a lumbar puncture. Histoplasma, blastomycosis, and HIV antibodies in cerebrospinal fluid were negative. Serum Cryptococcus antigen, Bartonella Hensale, and Bartonella Quintana IgM and IgG and rapid plasma reagin (RPR) tests were negative. Bone marrow acid-fast bacteria (AFB) stain and culture, C-ANCA, and P-ANCA were negative. 1, 25 OH, 25 Oh vitamin D3, and angiotensin-converting enzyme level were within normal limits. He underwent a left-sided frontal open brain biopsy, and pathology results were compatible with non-caseous granulomatous inflammation (Figure 2). Fungus tissue culture, sputum, and blood cultures were negative. He was empirically started on anti-tuberculous treatment with Ethambutol, Isoniazid (INH), Rifampin, Pyrazinamide, Pyridoxine, and dexamethasone. We were unable to achieve significant clinical improvement two weeks after anti-tuberculous treatment. Considering the possible diagnosis of neurosarcoidosis, methylprednisolone 1 g daily treatment was initiated for three days and gradually tapered. Later on, after six weeks, the culture of the brain biopsy result came positive for tuberculosis. Anti-tuberculosis (TB) medications were re-initiated, to be continued for 18 months. After two months of treatment, the control MRI brain with gadolinium contrast showed near resolution of the midline shift and a decrease in the left frontal lobe and bilateral parietal lobes edema (Figure 3).

## 3. Discussion

Intracranial tuberculomas are uncommon manifestations of tuberculosis, with only 0.2% of all biopsied brain masses account for tuberculomas. Sethi et al. reported that extra-pulmonary tuberculosis accounts for approximately 10–15% of all tuberculosis cases [8]. Intracranial tuberculomas can present with a seizure, headache, signs of intracranial hypertension, or focal neurologic symptoms. Neurologic deficits are a contributing factor based on the topographic location of the lesions [5]. Our patient presented with progressive weakness, headache, and confusion; he had no constitutional symptoms of fever, chills, night sweats, or productive cough. Neuroimaging findings of tuberculomas may demonstrate a solitary lesion or multiple contrast-enhancing lesions with extensive perilesional edema. A definitive diagnosis of tuberculoma can be confirmed with acid-fast bacilli in the pathologic specimen or histologic confirmation of epithelioid-giant cell granuloma with caseating necrotic material supported by a positive culture result.

Tuberculous bacilli cannot always be detected in cerebrospinal fluid (CSF) and even in excised mass, which means that laboratory findings of infection may be absent. Therefore, the possibility of infection cannot rule out from the negative results of bacterial culture [9]. In our patient, AFB smear of the blood and bone marrow AFB culture were both negative. Rheumatologic and infectious workup of the blood tests and CSF were unremarkable. MRI findings of intracranial tuberculomas may favor caseating or noncaseating granuloma. An iso-intense or hypo-intense appearance on T1-weighted MR images and hypo-intense appearance on T2-weighted images would indicate a solid caseating granuloma, and these lesions typically show ring enhancement. The appearance of non-caseating granulomas is hypo-intense on T1-weighted MR images and hyper-intense on T2-weighted images. There is a significant overlap with other intracranial focal lesions such as fungal granulomas, primary, and metastatic neoplasms.

Stereotactic brain biopsy is preferred in asymptomatic patients in whom an intracranial mass is suspected to be non-neoplastic. The diagnostic efficacy of the stereotactic brain biopsy is 85% when followed by paraffin sectioning and histopathological examination [3]. Open brain biopsy may be considered for a definitive diagnosis in controversial results [10]. In our patient, stereotactic brain biopsy results were inconclusive with showing reactive astrocytosis and the possibility of a non-caseating granuloma. In the literature, misdiagnosis of tuberculomas as sarcoidosis and vice versa have been described [11,12]. Atypical presentation of both diseases can lead to a misdiagnosis. The diagnosis of sarcoidosis requires relevant clinical features, pathological findings, and no possibility of an alternative diagnosis. Although non-caseating granuloma is compatible with sarcoidosis, the diagnosis cannot be made with 100% certainty without ruling out the alternative granulomatous diseases. In our case, the diagnosis was unclear between neurosarcoidosis and tuberculoma; finally, the brain tissue culture-confirmed brain tuberculoma. However, tuberculosis cultures generally result in up to 6 weeks. In the presence of high clinical and epidemiological risk factors, prophylactic anti-tuberculous medications can be initiated.

To date, intracranial tuberculomas remain a clinical challenge due to their rarity, non-specific presentation, and radiological findings. According to the current literature, up to 14% of brain tuberculomas can present with non-caseating granulomas [6]. Patients with brain tuberculoma have been traditionally treated with an anti-tuberculosis regimen starting with four medications; surgical intervention is rarely needed due to the high risk of iatrogenic tuberculous meningitis and even death. Surgical intervention in a patient with CNS tuberculoma is indicated in patients with acute symptoms due to increased intracranial pressure or in those with imaging that shows a persistent or enlarging mass after at least three months of anti-TB medication. The treatment response is monitored by CT Head or MRI Brain. The resolution of any enhancing lesion or enhancing lesion less than 1 cm or the presence of non-enhancing calcified residue can be considered as a resolution. We achieved near total resolution after two months of anti-tuberculous treatment.

## 4. Conclusions

The diagnosis of brain tuberculoma can be challenging due to atypical presentation and inconclusive pathological results. When tuberculosis is differential, a quantiferon gold test, advanced neuroimaging studies, pathological sampling, and cultures should be obtained. This report highlights the importance of including intracranial tuberculoma in the differential diagnosis of cerebral space-occupying lesions and for physicians to consider empiric anti-TB treatment for high-risk patients.
